# Early Life in a Barren Environment Adversely Affects Spatial Cognition in Laying Hens (*Gallus gallus domesticus*)

**DOI:** 10.3389/fvets.2015.00003

**Published:** 2015-03-18

**Authors:** Fernanda M. Tahamtani, Janicke Nordgreen, Rebecca E. Nordquist, Andrew M. Janczak

**Affiliations:** ^1^Animal Welfare Research Group, Department of Production Animal Clinical Science, Faculty of Veterinary Medicine and Biosciences, Norwegian University of Life Sciences (NMBU), Oslo, Norway; ^2^Emotion and Cognition Group, Department of Farm Animal Health, Utrecht University, Utrecht, Netherlands

**Keywords:** laying hen, chicken, cognition, spatial cognition, rearing, development, behavior

## Abstract

Spatial cognition in vertebrates is adversely affected by a lack of environmental complexity during early life. However, to our knowledge, no previous studies have tested the effect of early exposure to varying degrees of environmental complexity on specific components of spatial cognition in chickens. There are two main rearing systems for laying hens in the EU: aviaries and cages. These two systems differ from one another in environmental complexity. The aim of the present study was to test the hypothesis that rearing in a barren cage environment relative to a complex aviary environment causes long-lasting deficits in the ability to perform spatial tasks. For this purpose, 24 white Dekalb laying hens, half of which had been reared in an aviary system and the other half in a conventional cage system, were tested in a holeboard task. Birds from both treatment groups learnt the task; however, the cage-reared hens required more time to locate rewards and had poorer levels of working memory. The latter finding supports the hypothesis that rearing in a barren environment causes long-term impairment of short-term memory in chickens.

## Introduction

Animals must be able to perceive, store, and retrieve information in order to navigate their environment and maximize the ratio of benefits to costs. Birds should have good spatial cognition, allowing them to remember specific routes and landmarks so as to optimally utilize resources such as food, water, perches, and nests. They also need to use their knowledge of routes and landmarks effectively to escape potentially dangerous situations such as attacks that may have fatal consequences. Spatial learning and memory are, therefore, important for the fitness and survival of mobile species living in a complex environment. However, developing and maintaining cognitive ability is likely to be costly with regards to the energy required for neurogenesis and establishment of neural pathways (1–5). Natural and artificial selection are likely to favor individuals that program the allocation of resources to cognitive function, depending on the environment encountered during the early stages of development. These arguments emphasize the ultimate mechanisms underlying developmental plasticity, as suggested by the predictive adaptive response hypothesis (6). A poor environment during early life may also incur costs simply because of a lack of the stimulation necessary for optimal development, as suggested by the “silver spoon” hypothesis (7, 8). Both lines of argumentation suggest that early life in a simple environment should produce individuals with reduced cognitive ability compared to those raised in a more complex environment. Evidence of positive effects of enriched environments on solving cognitive tasks is available from previous studies in birds (9), rodents (10), and fish (11). On this basis, one would predict that birds exposed to a barren environment during early life would have poorer memory capacity.

Conventionally, laying hens in the EU are raised on specialized rearing farms to 15–18 weeks, at which time they are delivered to a specialized producer. They begin producing eggs between 18 and 22 weeks of age and are killed at 72–80 weeks of age, making space for a new set of birds. There are two major rearing systems for laying hens in the EU: aviary systems and cage systems. These two systems differ substantially from one another in, among other factors, environmental complexity. Rearing cages are barren environments containing 25 birds with access to food, water, and perches. Movement is restricted due to cage size. In the aviary-rearing system, at least 15,000 chickens are kept inside a large barn and are able to move both horizontally and vertically within it. Food, drinking nipples, and perches are available in specific locations on platforms elevated above the floor, and the chickens must navigate in order to access these. Differences in the early rearing environment have been shown to cause pronounced and long-lasting effects on spatial skill in domestic chickens (12, 13). However, to our knowledge, no previous studies have tested the effect of early exposure to varying degrees of environmental complexity on specific components of spatial cognition in this species. During the first 8 weeks of life, chickens reared from hatching with access to perches locate an elevated food reward faster than those reared without when tested at 16 weeks of age (13). In addition, the early rearing environment may influence the prevalence of floor eggs and cloacal cannibalism, possibly through its effects on spatial cognition (12). It is therefore likely that birds reared in barren cages will have a long-lasting deficit in ability to perform a spatial task compared to birds reared in a complex aviary system.

To test whether rearing in these different environments influences spatial cognition in laying hens with the same genetic background, a holeboard task was used (14). The task quantifies spatial discrimination learning, using the individual bird’s foraging behavior (15). It can be used to quantify working memory, general working memory, and reference memory under different conditions following habituation to the test arena. The holeboard is a maze in which food rewards can be found in a subset of potential sites. It is performed in a series of phases, during which certain conditions of the task are changed. This is relevant as the different conditions may influence the performance of the subjects. Using this methodology therefore facilitates testing predictions that the performance of birds reared in a barren environment are more adversely affected by the introduction of novel cues or a new reward configuration than those reared in a more complex environment. Working memory can be operationalized as the ratio of rewarded visits to the number of visits to baited holes, and reflects the chickens’ ability to avoid revisiting the baited set of holes within a trial (16, 17). The operational definition of general working memory is the ratio of the number of unique holes visited to the total number of visits to holes. It reflects the chickens’ ability to avoid holes that they have already visited during the trial (18), independent of whether baited or not.

Both forms of working memory contain information that is trial dependent, and are thought to be forms of short-term memory (19, 20). Reference memory is operationalized as the ratio of the number of visits to baited holes to the number of visits to all holes. This ratio indicates the chickens’ ability to distinguish between baited and unbaited holes (16, 17). Reference memory stores more general information about the task itself, such as the fact that food can be found at specific sites within the maze and how to access these food rewards. This latter type of information is thought to be trial independent and therefore stored as long-term memory (19, 20). Both short-term and long-term memories are necessary in solving a spatial task, and both are likely to require the allocation of resources for neurogenesis and the establishment of neural pathways. On the basis of the hypothesis that rearing in a barren cage environment compared to a complex aviary environment causes long-lasting deficit in the ability to perform a spatial task, we therefore predicted that cage-reared hens would have poorer measures of working memory, general working memory, and reference memory than aviary-reared hens. In addition to testing the above-mentioned hypothesis, we calculated correlations between response variables in order to describe the relationship between indices related to holeboard performance [see Ref. (14)].

To our knowledge, this is the first time a spatial holeboard task (14, 15) has been used to assess the effect of early exposure to varying environmental complexity in birds. The chicken is a highly relevant model organism for avian research that encompasses both basic and applied questions, warranting studies of environmentally determined developmental processes influencing cognitive ability.

## Materials and Methods

### General description of subjects and housing

Non-beak trimmed, female white Dekalb chickens (*Gallus gallus domesticus*) of up to 23 weeks of age and normal health were used in this study. These birds were hatched at a commercial hatchery and then reared in separate corridors in a single room until 16 weeks of age. Each corridor had either a cage or an aviary-rearing system. The house was 60 m x 20 m and contained 52,000 chickens in total. At 16 weeks of age, 24 birds from each treatment (48 birds in total) were transported 448 km by car in transport crates to the experimental facilities at the Norwegian University of Life Sciences campus Adamstuen, Oslo. Here, they were group-housed in Victorsson T10 furnished cages measuring 120 cm x 83 cm x 63 cm (length x height x width). Each cage contained dust-bathing substrate (powdered feed) on an elevated platform over the nest boxes (1554 cm^2^), two nest boxes, and two parallel perches (17 cm apart and 34 cm above the floor). Each cage contained two aviary-reared and two cage-reared birds. The cages at the experimental facilities were tiered within the house, creating two levels.

### Lighting and feeding

All the birds were exposed to the same light intensity, light schedule, and temperature, as recommended by the General Management Guide for White Dekalb Commercial Layers (21). During rearing, they were provided with *ad lib* access to both feed, using a chain dispersal system, and water. The feed type was conventional pullet feed produced and sold by Felleskjøpet, Norway (“Kromat oppdrett 1” for 0- to 6-week-old birds; “Kromat avl egg 1” for 6- to 8-week-old birds; and “Kromat oppdrett 2” for 8- to 15-week-old birds). At the experimental facility in which the adult hens were housed, a light-darkness cycle operated in accordance with recommendations by the Dekalb Management Guide (21). Feed was provided *ad lib*, using a feed trough at the front of the cage, and water was provided *ad lib* by nipple drinkers at the back of the cage (three per pen). Adult birds were manually fed Fjør Oppdrett Lett (Felleskjøpet) from a feed trough outside the front of the cage until start of lay (16- to 18-week-old birds), and Fjør Egg (Felleskjøpet) until the end of the experiment (24-week-old birds).

### Rearing treatments

**Figure 1:**
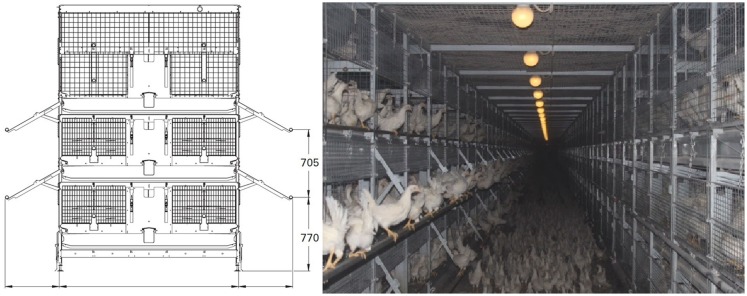
**A schematic representation and photograph of one unit of the housing system viewed from the end of the row and the corridor, respectively**. In the schematic representation, aviary rows or cages are on either side of a corridor. Perches are shown as circles. Measurements are provided in mm. Modified with permission from Big Dutchman. The left side of the photograph and the floor represent the conditions for aviary-reared birds. The right side of the photograph indicates the conditions for cage-reared birds, apart from the fact that birds were not housed in the third tier. In the present study, cage- and aviary-reared birds faced separate corridors. The width of corridors was 2 m.

All the birds were housed in a single room in a Natura Primus 1600 system (Big Dutchman; Figure 1)^1^, designed for aviary rearing of laying pullets. This system consists of cages stacked in three tiers on either side of a corridor, allowing inspection by the caretaker. The cage dimensions are 120 cm x 60 cm x 80 cm (length x height x width). Each aviary cage contains a 120 cm feed trough, one 120 cm perch, and five drinking nipples. All cages can be opened at the front so that the birds can move between each tier and the floor of the corridor. Ramps run from the floor to the second tier to increase the pullets’ ease of access. When the cage doors are open, perches extend from the front of the first and second tiers. The density was 25 birds/m^2^ during the first 4 weeks of life for both treatments. After cage doors were opened, the density of aviary-reared birds was reduced to 12 birds/m^2^ when taking account of the sum of floor space in aviary tiers and the hallway.

Upon delivery to the rearing farm, immediately after hatching, all the chicks were initially placed in cages on the first and second tiers. At 4 weeks of age, the aviary-reared birds (half the birds in the house) were released from their cages by opening the doors, allowing them to move between the corridor floor and each aviary tier on either side of the corridor, until the end of the rearing phase at 16 weeks. Meanwhile, the cage-reared birds (the remainder) were kept in cages on the first and second tiers. The aviary-reared and the cage-reared birds were housed in separate corridors throughout the rearing phase.

### Principles of the holeboard test

The holeboard test can be used to quantify working memory, general working memory, and reference memory under different conditions following habituation to the test arena. An initial acquisition phase is used to test the birds’ ability to learn the location of baited cups without the provision of specific cues. A second cued phase involves the addition of novel cues associated with baited cups. Following the first trial during which neophobic responses may be observed, the cued phase introduces additional information that may improve cognitive performance relative to performance in the uncued task. This is followed by a third over-training phase in which cues are again removed in order to re-establish scores for baseline performance. The fourth reversal phase involves testing the birds’ ability to learn the location of rewarded cups after the introduction of a new uncued configuration. This last phase introduces a change that requires birds to replace previous information regarding the configuration of rewarded cups with information about the new configuration.

### Holeboard design

**Figure 2:**
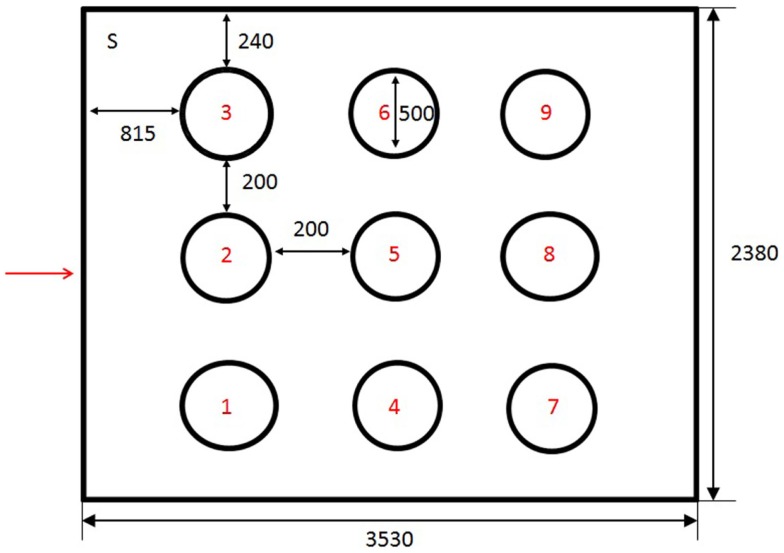
**Schematic representation of holeboard arena**. Holes were numbered from 1 to 9. Chickens were introduced into the holeboard from the corner of the room marked S. The entrance to the arena is marked with an arrow. The dimension of the test arena was 3530 mm × 2380 mm. Circles were 240 mm from the long wall and 815 mm from the short wall. They had a diameter of 500 mm and were separated by a 200 mm gap.

The holeboard test comprised a modification of methods described by Nordquist et al. (14) (Figure 2). The holeboard arena was an arena measuring 2.38 m x 2.38 m. The walls were concrete and the doors were steel, providing visual and limited auditory isolation from adjacent rooms containing the home pens and the observer, respectively. The arena contained nine chalk circles, each with a diameter of 50 cm. The circles were distributed in a 3 × 3 matrix in the arena. Inside each circle rested a plywood surface (19 cm x 19 cm) with a small blue cup positioned in the center. A bird was considered to have visited a cup if it crossed into the chalk circle surrounding a cup. The distance between each cup was 70 cm. The holeboard was swept clean between trials and the chalk circles redrawn if necessary. The behavior of the chickens was recorded using MSH-Video (M. Shafro & Co.)^2^ from a computer screen attached to a video camera set up above the holeboard arena.

### Timeline and overview of procedures for habituation and testing in the holeboard

The birds were habituated to the cups over 7 days, starting upon arrival at the experimental facilities at 16 weeks old. They were then habituated to the holeboard apparatus over 5 days starting at 17 weeks old. Following habituation, the birds were trained and tested individually in two trials per day over 28 working days (14). This was divided into the following phases: an uncued acquisition phase starting at 18 weeks (28 trials over 14 test days); a cued acquisition phase starting at 21 weeks (10 trials over 5 test days); an over-training phase starting at 22 weeks (10 trials over 5 test days); and a reversal phase starting at 23 weeks (8 trials over 4 test days).

### Habituation and selection of experimental birds

All 48 birds (24 from the aviary-rearing treatment and 24 from the cage-rearing treatment) were habituated to the blue cups that contained rewards in the holeboard, and trained for a week to associate the cups with a mealworm reward by provision of live mealworms in the cups in the home pen three times daily. Next, these birds were allowed to habituate to the holeboard test arena for five consecutive days in daily sessions of 5 min. During the first habituation session, the birds were exposed to the apparatus in pairs of the same rearing treatment; for the remainder of the habituation, training, and testing sessions, the birds were exposed to the arena alone. During habituation sessions, all nine cups contained one mealworm. The habituation sessions were terminated when each bird had found and eaten all nine mealworms or 5 min had elapsed, whichever occurred first. According to a pilot study performed by our group, 33% of chickens fail to find any mealworms after several training days. A subset of 24 birds (*n* = 12 per treatment; one bird per treatment per cage) was therefore selected for use in the testing phase. The criterion for selecting which bird should be used for testing was based on performance during habituation, such as exploring the room and finding and eating mealworms. After the 5 days of habituation, the better of the two chickens of a given treatment from a given cage was chosen. If there was no clear difference between the two chickens, one was chosen at random by drawing numbered papers from a jar.

### General procedures for the uncued acquisition phase

During holeboard training and testing, three cups were baited with worms and the remaining six left empty. The configuration refers to the spatial position of the baited cups in the arena. Six configurations were randomly chosen and four chickens were randomly assigned to each configuration. The order in which different birds were tested on a given day was randomized. The chickens were trained to find the three baited cups among the nine cups in the holeboard without any specific cues to guide them. The mealworms were only visible to birds after they had chosen a cup by entering the circle surrounding it. At the start of each test, the chicken was placed in the top left corner of the holeboard (Figure 2) and the experimenter quickly left the room. The 24 chickens were tested in two separate trials per day. The same random test order was used for both trials on the same day. Chickens were returned to their cage between trials.

### Procedures specific to each training and test phase

For uncued acquisition training on test days 1–14 (trial 1–28), the same configuration of baited cups was used for each bird. During cued acquisition on test days 15–19 (trials 29–38), extra cues were added to the three baited cups in the form of a colored plywood base (red instead of the normal light wood color) without changing the configuration of baited cups. For over-training on test days 20–24 (trials 39–48), the baited cups were returned to their uncued form and the birds were further trained (over-training) in the uncued format of the holeboard. In the reversal phase on test days 25–28 (trials 49–56), the chickens were trained to find mealworms in a new configuration of baited, uncued, cups.

### Ethical statement

This experimental work was approved by the Institutional Animal Care and Use Committee at NMBU under ID number 6189.

### Data processing and statistical analysis

#### Holeboard parameters

The following measures were noted and/or calculated for each trial. Trial duration was defined as the total duration until all the mealworms had been eaten, or the maximum of 5 min had elapsed. Working memory was defined as the ratio of rewarded visits to the number of visits to the baited holes. This ratio reflects the chickens’ ability to avoid re-visits to the baited set of holes within the trial (16, 17). General working memory was defined as the ratio of the number of unique holes visited to the total number of hole visits. This ratio reflects the chickens’ ability to avoid holes already visited within the trial (22). Reference memory was defined as the ratio of the number of visits to baited holes to the number of visits to all holes. This ratio indicates the chickens’ ability to discriminate between baited and unbaited holes (16, 17). For each individual, the average of each of the four measures (trial duration, working memory, general working memory, and reference memory) was calculated per phase, and this average score was used for statistical analysis. An exception was made for the calculation of correlations on the basis of raw scores.

#### Effects of rearing treatment and phase

The effect of rearing environment (treatment) on the four parameters described above was tested in a repeated measures ANOVA, with bird as random factor nested in treatment, and treatment and phase as fixed factors. The interaction between treatment and phase was included in the model. Phase (uncued acquisition, cued acquisition, over-training, and reversal) was the repeated factor. The trial duration data did not fulfill all of the assumptions of ANOVA (equality of variance and normality of residuals), and was therefore transformed using a Box–Cox transformation. Where significant interactions were found, the data were subjected to a *post hoc* Student’s *t*-test comparing treatment means within phase, resulting in a total of four comparisons. The critical *p*-value associated with these *post hoc t*-tests was Bonferroni corrected to *p* = 0.0125. Following ANOVA indicating a main effect of phase, *post hoc* comparison of phase means was performed using the Tukey’s test (Tukey’s HSD test). Pearson correlation coefficients between each pair of memory indices within each trial of the uncued acquisition, cued acquisition, over-training, and reversal phases were calculated in order to describe associations between them for comparison with previous studies. Although all correlations were calculated for the sake of completeness, our focus was on the relationship between the conceptually independent indices of working memory and reference memory. In addition, Pearson correlation coefficients between working memory and Box–Cox transformed trial duration values in each trial of the reversal phase were calculated. This was done after identifying an effect of the rearing treatment during this phase on both trial duration and working memory in order to describe the association between the two. The statistical software was JMP^®^ 11.1.1 (SAS Institute Inc.).

## Results

### General information

**Figure 3:**
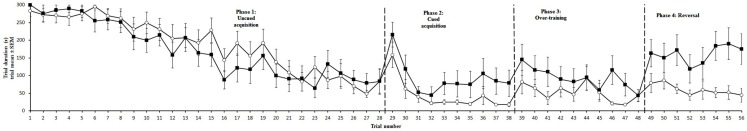
**Trial duration for aviary (∘) and cage-reared (■) chickens in the holeboard task**. Trial duration is presented as mean and standard error of the mean (SEM). Dashed lines mark the transitions between uncued, cued, over-training, and reversal phases.

**Figure 4:**
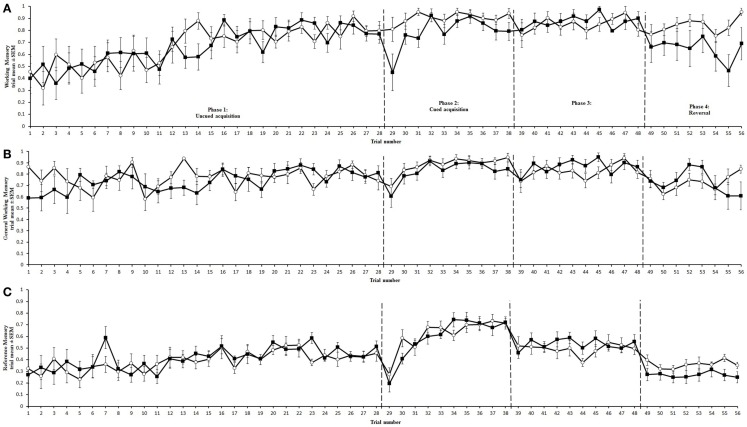
**Memory performance of aviary (∘) and cage-reared (■) chickens in the holeboard task**. Working memory **(A)**, General working memory **(B)**, and reference memory **(C)** are presented as mean and standard error of the mean (SEM). Dashed lines mark the transitions between uncued, cued, over-training, and reversal phases.

Two chickens from the cage-reared treatment and one from the aviary-reared treatment did not search for bait in the holeboard, despite extensive training. Their data were excluded from the statistical analyses, reducing the number of individuals in the cage and aviary-reared treatments to 10 and 11, respectively. Mean values for trial duration, working memory, general working memory, and reference memory for each treatment during each holeboard phase are presented in Table 1 and mean values for each trial are shown in Figures 3 and 4 for trial duration and cognitive parameters, respectively. Statistics from the holeboard (*F*- and *p*-values) are presented in Table 2, apart from *post hoc* tests, which are provided in the text. Correlations between memory indices are presented in Table 3, and correlations between working memory and trial duration in the reversal phase are presented in Table 4.

**Table 1:** **Mean and standard error of the mean (±SEM) values for trial duration, working memory, general working memory, and reference memory for aviary- and cage-reared birds in the four training phases of the holeboard task**.

	Aviary		Cages	
	Mean	±SEM	Mean	±SEM
**Trial duration (s)**
Uncued acquisition	187.23	6.89	172.57	7.24
Cued acquisition	43.06	6.61	92.95	11.43
Over-training	52.02	7.18	93.19	11.23
Reversal	**59.95**	7.02	**161.21**	14.51
**Working memory**
Uncued acquisition	0.67	0.02	0.66	0.02
Cued acquisition	0.90	0.016	0.79	0.027
Over-training	0.85	0.019	0.87	0.019
Reversal	**0.84**	0.02	**0.65**	0.045
**General working memory**
Uncued acquisition	0.77	0.014	0.74	0.016
Cued acquisition	0.88	0.015	0.83	0.021
Over-training	0.83	0.019	0.87	0.017
Reversal	0.73	0.021	0.73	0.03
**Reference memory**
Uncued acquisition	0.39	0.013	0.41	0.013
Cued acquisition	0.62	0.022	0.59	0.024
Over-training	0.50	0.018	0.54	0.017
Reversal	0.36	0.014	0.27	0.018

*Mean values that differ significantly between treatments within phase are marked in bold*.

**Table 2:** **Results of repeated measures ANOVA testing effects of treatment (cage or aviary rearing) and phase (uncued acquisition, cued acquisition, over-training, and reversal) in the holeboard task**.

Parameter	Statistics (*F*- and *p*-values)		
	Treatment	Phase	Treatment x phase
Trial duration	*F*_1,19_ = 3.361, *p* = *0.082*	*F*_3,57_ = 25.44, ***p****** < 0.0001***	*F*_3,57_ = 3.897, ***p****** = 0.013***
Working memory	*F*_1,19_ = 3.04, *p* = *0.097*	*F*_3,57_ = 9.12, ***p****** < 0.0001***	*F*_3,57_ = 2.75, ***p****** = 0.051***
General working memory	*F*_1,19_ = 0.136, *p* = 0.716	*F*_3,57_ = 12.06, ***p****** < 0.0001***	*F*_3,57_ = 1.116, *p* = 0.350
Reference memory	*F*_1,19_ = 0.203, *p* = 0.657	*F*_3,57_ = 28.38, ***p****** < 0.0001***	*F*_3,57_ = 1.545, *p* = 0.213

*Trends (0.100 ≥ *p* > 0.051) are italicized. Values considered significant are in bold (*p* ≤ 0.051)*.

**Table 3:** **Pearson correlation coefficients and associated *p*-values for correlations between working memory, general working memory, and reference memory**.

Trials	Working memory– general working memory		Working memory– reference memory		General working memory– reference memory	
	*r*	*p*	*r*	*p*	*r*	*p*
**Uncued acquisition**	
1	0.1282	0.579	0.2791	0.220	0.1310	0.571
2	0.1801	0.435	**0.4911**	**0.024**	0.1700	0.463
3	0.1569	0.497	**0.6259**	**0.002**	*0.3929*	*0.078*
4	**0.6325**	**0.002**	**0.6983**	**0.0004**	**0.6603**	**0.001**
5	0.1075	0.643	**0.5574**	**0.009**	0.1246	0.590
6	**0.6118**	**0.003**	**0.7232**	**0.0002**	**0.6519**	**0.001**
7	**0.5012**	**0.021**	0.0747	0.748	0.2212	0.335
8	0.1700	0.461	**0.6979**	**0.0004**	0.1130	0.626
9	0.2430	0.288	**0.8873**	**0.0001**	0.1988	0.388
10	**0.5442**	**0.011**	**0.6778**	**0.0007**	*0.4181*	*0.059*
11	−0.0438	0.850	**0.5190**	**0.016**	−0.2016	0.381
12	0.3346	0.138	0.2325	0.310	**0.5222**	**0.015**
13	*0.3948*	*0.076*	**0.6316**	**0.002**	0.0197	0.932
14	**0.8440**	**0.0001**	0.2856	0.209	**0.5813**	**0.006**
15	**0.8394**	**0.0001**	0.3597	0.109	0.3587	0.111
16	**0.4409**	**0.045**	**0.5679**	**0.007**	0.3488	0.121
17	**0.7689**	**0.0001**	0.2133	0.353	**0.6476**	**0.001**
18	**0.9286**	**0.0001**	**0.5848**	**0.005**	**0.7550**	**0.0001**
19	**0.6769**	**0.0008**	0.2221	0.333	**0.5861**	**0.005**
20	**0.8834**	**0.0001**	**0.4482**	**0.042**	**0.4826**	**0.027**
21	**0.9742**	**0.0001**	*0.4020*	*0.071*	**0.4387**	**0.047**
22	**0.8533**	**0.0001**	0.3237	0.152	**0.4853**	**0.026**
23	**0.7065**	**0.0003**	**0.5285**	**0.014**	**0.7024**	**0.004**
24	**0.6087**	**0.003**	0.1380	0.551	**0.6547**	**0.001**
25	**0.5632**	**0.008**	**0.7322**	**0.0002**	*0.4227*	*0.056*
26	**0.8040**	**0.0001**	−0.0720	0.756	0.2646	0.246
27	**0.9159**	**0.0001**	0.1707	0.459	0.3299	0.144
28	**0.9016**	**0.0001**	**0.5454**	**0.011**	**0.6636**	**0.001**



**Cued acquisition**	
29	**0.4385**	**0.047**	**0.6563**	**0.001**	**0.6408**	**0.002**
30	**0.8728**	**0.0001**	*0.3850*	*0.085*	**0.5637**	**0.008**
31	**0.7531**	**0.0001**	0.0673	0.771	**0.4481**	**0.042**
32	**0.8861**	**0.0001**	0.0596	0.797	0.2325	0.310
33	**0.9314**	**0.0001**	0.1087	0.039	0.0781	0.737
34	**0.8964**	**0.0001**	0.1964	0.393	*0.4055*	*0.068*
35	**0.9137**	**0.0001**	**0.5104**	**0.018**	**0.6729**	**0.0008**
36	**0.8232**	**0.0001**	**0.5572**	**0.009**	**0.4635**	**0.034**
37	**0.9816**	**0.0001**	**0.5981**	**0.004**	**0.5988**	**0.004**
38	**0.9689**	**0.0001**	*0.3885*	*0.082*	*0.3877*	*0.0825*



**Over-training**	
39	**0.9138**	**0.0001**	0.6320	0.002	**0.7781**	**0.0001**
40	**0.9574**	**0.0001**	*0.3719*	*0.097*	**0.4683**	**0.032**
41	**0.9100**	**0.0001**	*0.4010*	*0.072*	**0.6313**	**0.002**
42	**0.9132**	**0.0001**	**0.4341**	**0.049**	**0.5678**	**0.007**
43	**0.9035**	**0.0001**	0.1750	0.448	**0.4407**	**0.045**
44	**0.8935**	**0.0001**	0.3039	0.180	**0.4476**	**0.042**
45	**0.8142**	**0.0001**	**0.5244**	**0.015**	**0.7318**	**0.0002**
46	**0.9002**	**0.0001**	**0.5133**	**0.017**	**0.6171**	**0.003**
47	**0.6910**	**0.0005**	0.1844	0.424	0.3677	0.101
48	**0.8906**	**0.0001**	**0.5138**	**0.017**	**0.5605**	**0.008**
**Reversal**	
49	**0.5112**	**0.018**	**0.5478**	**0.010**	*0.3984*	*0.074*
50	0.2342	0.307	0.1387	0.548	*0.3973*	*0.075*
51	**0.5122**	**0.018**	**0.6420**	**0.002**	**0.4808**	**0.027**
52	0.0524	0.821	**0.6713**	**0.001**	0.1836	0.426
53	0.0304	0.896	**0.4989**	**0.021**	0.2409	0.293
54	**0.5664**	**0.007**	**0.5434**	**0.011**	0.3604	0.108
55	**0.7575**	**0.0001**	**0.7721**	**0.0001**	**0.5128**	**0.0175**
56	**0.8992**	**0.0001**	**0.6520**	**0.0001**	**0.8531**	**0.0001**

*Correlations with an associated *p*-value < 0.05 are in bold. Correlations with 0.10 > *p* > 0.05 are italicized*.

**Table 4:** **Pearson correlation coefficients and associated *p*-values for correlations between working memory and trial duration during the reversal phase (trials 49–56)**.

Trial	Working memory-trial duration (Box–Cox transformed)	
	*r*	*p*
49	**−0.6831**	**0.0006**
50	−0.3654	0.103
51	**−0.5714**	**0.007**
52	**−0.8354**	**0.0001**
53	**−0.6135**	**0.0003**
54	**−0.7086**	**0.0003**
55	**−0.7900**	**0.0001**
56	**−0.6424**	**0.002**

*Correlations with an associated *p*-value <0.05 are in bold*.

### Phase effects and correlations between indices

Trial duration and all memory parameters (Table 1) were significantly affected by phase (Table 2). Mean trial duration decreased during the cued acquisition, over-training, and reversal phases relative to the uncued acquisition phase (Tukey’s test *p* < 0.0001 for all comparisons; Figure 3). Working memory increased from the uncued acquisition to the cued acquisition and over-training phases (Tukey’s test *p* = 0.0006 and *p* = 0.0002, respectively; Figure 4A). General working memory performance for both aviary- and cage-reared chickens increased from the uncued acquisition phase to cued acquisition and over-training phases (Tukey’s test *p* = 0.002 and *p* = 0.005, respectively; Figure 4B). It then decreased in the reversal phase, returning to uncued acquisition levels (Tukey’s test *p* < 0.0001). Reference memory increased from uncued acquisition to cued acquisition (Tukey’s test *p* < 0.0001; Figure 4C). It then decreased from cued acquisition to the over-training phase (Tukey’s test *p* = 0.042) and from over-training phase to the reversal phase (Tukey’s test *p* < 0.0001).

Correlations between working memory and general working memory were mostly weak to moderate during the first half of the acquisition phase, and most of the reversal phase, when performance was not at its peak (Table 3). Correlations were higher during the latter half of the acquisition phase as well as the cued phase and over-training phases, when performance was better. Correlations between working memory and reference memory were higher during the first half of the acquisition phase and reversal phase when performance was poorer, and lower during the latter half of the acquisition phase as well as during cued acquisition and over-training phases when memory performance was better. Correlations between working memory and trial duration were negative and mostly high throughout the reversal phase (Table 4).

### Treatment effects

There was no main effect of rearing treatment on any of the holeboard variables (Table 2). There were, however, significant interactions between treatment and phase for trial duration and working memory (Table 2). During the reversal phase, the cage-reared chickens took longer to complete the holeboard task than the aviary-reared chickens (*t* = 2.99; *p* = 0.0044). Furthermore, the aviary-reared chickens had better working memory during the reversal phase than cage-reared birds (*t* = −2.88; *p* = 0.0052).

## Discussion

### Summary

The results show the effects of the rearing environment on working memory and trial duration in a holeboard task, and support the hypothesis that rearing in a barren cage environment relative to a complex aviary environment causes long-lasting deficit in the ability of chickens to perform a spatial task. With the exception of the three birds that did not learn the task, the effects of phase indicated that the holeboard task was a valid approach to quantifying working memory, general working memory, and reference memory in laying hens. This was confirmed by higher average scores for working memory, general working memory, and reference memory during cued acquisition and over-training than during uncued acquisition. The present study also supports the previous finding with chickens (14) and pigs (23) that food deprivation may not be necessary if birds are provided with an attractive reward. The working memory performance of laying hens in the present study corresponds to previous reports, as working memory scores of 0.7–0.8 have been reported for chickens (14). These scores are directly comparable to those in the present study in which three of nine holes were rewarded. However, direct comparison of ratios across studies is problematic because of the variation in protocols used, such as variations in the number of holes, number of rewarded holes, and maze configuration. Nonetheless, scores closer to 1 indicate better performance, and scores closer to 0 indicate worse performance. With that caveat, the relatively high working memory scores in this study, in the range of 0.7–0.8, have also been reported for gerbils (24), rats (25), and mice (26, 27), while pigs often achieve a near-perfect performance in working memory (28, 29). Reference memory typically starts at low levels such as 0.3 in gerbils and rats, and 0.4 in chickens and mice (14, 24–26). Some studies report maximum reference memory values as high as 0.85 in mice (26) and pigs (28, 29), whereas others report comparatively low values of 0.4 in gerbils (24). The reference memory scores of 0.4–0.6 reported in the over-training phase fall at the lower end of this scale, which is strongly comparable to a previous study in chickens using the holeboard (14). This indicates that, while they are capable of learning the task, chickens do not show the high levels of reference memory performance observed in some other species.

### Treatment effects on cognition

Working memory is considered to be a form of short-term memory, while reference memory is considered to be a form of long-term memory (19, 20). Working memory contains elements that are trial dependent – “what has happened, when and where” such as which holes have been visited – and helps the bird avoid revisits and maintain an effective foraging strategy (15). It must therefore be reset after each trial so as not to influence performance in the next trial (30, 31). Cage-reared birds had lower levels of working memory than aviary-reared birds during the reversal phase. This difference in short-term working memory indicates that rearing in a barren environment adversely affects working memory. It is noteworthy that the housing in the same environment at the research facility and repeated training in a cognitive task for a 6-week period does not compensate for the cognitive deficit caused by early life in a relatively impoverished environment. A previous study indicates that the first 2 months of life without access to perches is enough to impair the cognitive skills necessary to move around a three-dimensional space in laying hens tested at 16 weeks of age (13). To our knowledge, there is only one previous study testing laying hens in a spatial holeboard [see Nordquist et al. (14)]. The reduction in working memory in cage-reared birds at the reversal phase, seen in conjunction with the corresponding elevated latency to eat all the mealworms, may indicate that these individuals are more sensitive to environmental change than aviary-reared birds.

Reference memory, as opposed to working memory, is memory of the general rules of the task, such as the fact that holes may or may not contain food rewards. It holds information that is relevant across several of the trials and is, therefore, trial independent (31, 32). Over the course of several trials during the uncued, cued, and over-training phases of the holeboard task, the chickens learnt that the food rewards were always in certain holes and committed these facts to reference memory, as part of the trial-independent rules of the task. This reference memory was, consequently, challenged in the reversal phase of the task. The present results, however, show no rearing treatment effects on reference memory.

In a typical aviary environment, chickens have ample opportunity to move in three-dimensional space and to perform a wide range of natural behaviors such as wing flapping, dust bathing, and flying. They also have both positive and negative contact with a large number of conspecifics. In the case of negative (antagonistic or aggressive) social interactions, a subordinate chicken has the option of moving away from the area to avoid or escape the attacker. The chickens must also be able to find food troughs, drinking nipples, nest boxes, and perches throughout the aviary.

In a furnished cage system, the chickens have very limited space in which to move. Some natural behaviors such as wing flapping and flying are difficult to perform. Vertical movement is limited to about 50 cm. Each hen normally has physical and social contact with 8–10 other hens, as for example, in the modified Victorsson T10 cages used in the present study. All resources available to each hen are within the cage and, therefore, the birds need not search for these. A caged laying hen’s environment may thus present her with cognitive challenges that are similar to the challenges met in the first three phases of the holeboard (uncued acquisition, cued acquisition, and over-training). The environment that a caged hen experiences is normally very stable. In this type of surroundings, reference memory is arguably the most relevant memory component, one that holds the general rules and facts about the environment on which the chickens can always depend. In the aviary system, however, each hen has the potential to find herself in a wider range of situations, both in terms of location and social interaction. In this case, working memory is likely to be valuable, as it allows the chicken to interpret stimuli based on each individual situation, and to navigate through a complex environment that may change depending on her location in the house and elevation above the floor. Overall, cognition is favored in environments with greater spatial variability than those that are stable (5). The results of the present study therefore suggest that the complexity of the aviary-rearing environment may encourage the development of short-term memory.

No rearing treatment effects on general working memory were found. General working memory is the ratio of the number of unique holes visited to the total number of hole visits. During the reversal phase, where treatment effects were found in working memory, general working memory for both treatments showed a similar decrease. In this phase, the configuration of cups was changed, forcing the chickens to explore more cups to find the food rewards. The reduction during this phase in working memory and general working memory for cage-reared birds, but only in general working memory for aviary-reared birds, indicates that cage-reared chickens revisited both baited and unbaited cups. In contrast, aviary-reared birds revisited only the cups that may have been baited in previous phases, and are now unbaited, thus reducing general working memory but not working memory. This further underpins the suggestion that aviary-reared birds have better short-term memory than cage-reared birds.

The previous discussion partly rests on the assumption that spatial working and reference memory are psychologically distinct. Correlational data and a lack of correspondence between treatment effects influencing one indicator but not the other, from studies in rats and mice, support the idea that they are independent [reviewed by van der Staay et al. (15)]. A previous study in chickens suggests that working and reference memory in this species may not be fully independent, as indicated by correlations between these indices, especially during the acquisition phase (14). The present study, indicating many moderate and high correlations between working and reference memory indices mainly in the early trials of uncued acquisition and the reversal phase, corroborates this and suggests links between these memory types for chickens mainly during test stages at which previous memory is challenged by introduction to testing or exposure to a new configuration of rewards. However, the observation that the rearing treatment influenced working memory but showed no tendency to influence reference memory suggests that these indices are functionally different also in chickens.

### Treatment effects on trial duration

The corresponding adverse effects of cage rearing on trial duration and working memory are interesting in view of questions raised by van der Staay et al. (15) regarding the relationship between these variables. They suggest that individuals taking longer to complete a task must bear the additional burden of retaining information stored as working memory for longer periods than those that do so more quickly. Indeed, the present study presents high negative correlations between working memory and trial duration during the reversal phase. Although it cannot be answered on the basis of data in the current study, this possibility raises the question of possible causal relationships between speed of task completion and working memory performance. The treatment effect on trial duration during the reversal phase suggests that aviary-reared birds may have a lower threshold for expressing appetitive behavior directed at the mealworm rewards. As appetitive behavior in chickens is likely to be mediated by activity in dopaminergic reward pathways (33), this suggests that rearing in an enriched environment may alter this system. Indeed, the Dopamine D2 antagonist Haloperidol adversely affects spatial learning and memory in rats (34, 35), as well as appetitive responses in chickens (33). A study using adult domestic chickens housed in a free-range system or battery cages indicated that free-range housing caused changes to the dopaminergic system in the dorsomedial hippocampus (36). Taken together, the current results, viewed in the context of related studies, therefore suggest that the complexity of the early rearing environment may influence the dopaminergic system in chickens.

### Proximate mechanisms

An aviary-rearing system, with its complexity and higher opportunity for novel situations, seems to prepare chickens to cope with new tasks by increasing their ability to retain short-term spatial and temporal information about the environment. This may be possible through increased neuroplasticity (37). Previous studies have shown an increase of hippocampal neuron density in mice housed in an enriched environment compared to mice reared in standard laboratory cages (38), and development of longer dendrites in hippocampal neurons in chicks reared with visual barriers, particularly in the right hemisphere (39). It is also possible that the physical challenges and opportunities provided by aviary systems (e.g., flying and perching at different levels) have a positive effect on neurogenesis, as physical skill training in rodents increases the number of surviving new cells in the hippocampus (40) and alters dopaminergic components of the hippocampus in chickens (36). In mice, postnatal environment and environmental change affects cognition, as measured by performance in a water maze learning task, and neurogenesis (41). Mice housed in an enriched environment for 8 weeks during the juvenile period showed better performance in the water maze and higher prevalence of newly generated neurons in the hippocampus than individuals housed in impoverished environments. Likewise, mice transferred from an impoverished environment to an enriched one displayed better water maze performance and higher hippocampal neurogenesis than those transferred from enriched environments to an impoverished one. Moreover, rats exposed to cognition-enhancing drugs (tacrine, nefiracetam, and deprenyl) showed an increase in neuroplasticity indistinguishable from the increase caused by environmental complexity (42).

### Variability in general working memory

General working memory levels were variable during the uncued acquisition phase of the holeboard task. As previously mentioned, working memory is a ratio of rewarded visits to the number of visits to the baited set of holes, while general working memory is the ratio of all holes visited to the total number of visits to any hole. This high degree of variability observed for general working memory may therefore indicate that chickens are better at remembering events when they have recent memory of both successful and failed attempts to find the food rewards. This suggests that the information the hen acquires from visiting an empty hole is as informative as the information acquired from a visit to a baited hole. During trials where the chickens performed well and visited only baited cups, the trial was terminated as soon as all mealworms had been found, so the chickens had no chance to explore the other cups. This could then result in poorer performance in the following trial as chickens had to explore other cups as well as the ones with mealworms. Indeed, a similar phenomenon has been observed in studies of spatial memory in other non-caching bird species, with authors suggesting a distinct effect of proactive interference, that is, the information about a rewarded site is influenced by the exploration of other sites prior to finding the reward (43, 44). Therefore, hens seem to have better recollection of where baited sites are located when they had explored both baited and unbaited sites in the previous trials. This illustrates that visits to unbaited holes also provide birds with relevant information that they store and retrieve when needed. It is possible that the birds continued to visit empty holes intermittently prior to completing the task, because the cost of checking empty holes may have been small relative to the potential benefit of detecting a change in reward contingency. With the introduction of the cues, and thereafter in the over-training phase, general working memory stabilized and remained high. This progression is also supported by a previous study indicating no difference between caching and non-caching species in memory acquisition when the food reward is visible (43). Accordingly, general working memory levels again began to oscillate in the reversal phase, indicating that the memory of the previous bait configuration was challenged and the birds had to create new memories.

## Conclusion

The results of this study support the hypothesis that rearing in a barren cage environment relative to a complex aviary environment causes long-lasting deficit in the ability to perform a spatial task, as indicated by effects on chickens’ working memory. Exposure to varying degrees of early environmental complexity thus influences how well birds remember the type of stimulus presented, when it was presented, and where this happened. Furthermore, the effects documented in the present study were rather long-term, as the last treatment effects were found over two months after birds were removed from the rearing environment.

## Author Contributions

FT participated in the design of the study, carried out data collection and data analysis, and drafted the manuscript; RN participated in the design of the study and drafted the manuscript; JN participated in the design of the study, carried out data analysis, and drafted the manuscript; AJ participated in the design of the study and drafted the manuscript. All authors approved the final manuscript.

## Conflict of Interest Statement

No conflicts of interests exist in regards to this study. The funding organizations the Foundation for Research Levy on Agricultural Products (FFL), the Agricultural Agreement Research Fund (JA), and Animalia (Norwegian Meat and Poultry Research Centre) finance applied agricultural research in collaboration with the private and public sectors. These parties’ sole interest in the present study was to support publication of the unbiased results in order to provide advice to poultry rearers.

## Footnotes

^1^http://www.bigdutchmanusa.com/

^2^www.guard.lv
